# Adjusting the Prerelease Gut Microbial Community by Diet Training to Improve the Postrelease Fitness of Captive-Bred *Acipenser dabryanus*

**DOI:** 10.3389/fmicb.2020.00488

**Published:** 2020-04-21

**Authors:** Haile Yang, Xiaoqian Leng, Hao Du, Jiang Luo, Jinping Wu, Qiwei Wei

**Affiliations:** Key Laboratory of Freshwater Biodiversity Conservation, Ministry of Agriculture and Rural Affairs of China, Yangtze River Fisheries Research Institute, Chinese Academy of Fishery Sciences, Wuhan, China

**Keywords:** reintroduction, captive-bred population, release cost, diet training, gut microbes, *Acipenser dabryanus*

## Abstract

As one of the most important tool for biodiversity restoration and endangered species conservation, reintroduction has been implemented worldwide. In reintroduction projects, prerelease conditioning could effectively increase postrelease fitness and survival by improving animals’ adaptation to transformation from artificial to natural environments. However, how early-life diet training affects individuals’ adaptation, fitness, and survival after release remains largely unknown. We hypothesized that early-life diet training would adjust the host’s gut microbial community, the gut microbial community would influence the host’s diet preference, and the host’s diet preference would impact its adaptation to diet provision transformation and then determine postrelease fitness and survival. To verify this hypothesis, we investigated the growth characteristics and gut microbes of Yangtze sturgeon (*Acipenser dabryanus*) trained with natural and formula diets at both the prerelease and postrelease stages. The results showed that (1) the gut microbial communities of the individuals trained with a natural diet (i.e., natural diet group) and formula diet (i.e., formula diet group) evolved to the optimal status for their corresponding diet provisions, (2) the individuals in the natural diet group paid a lower cost (i.e., changed their gut microbial communities less) during diet transformation and release into the natural environment than did the individuals in the formula diet group, and (3) the gut microbes in the natural diet group better supported postrelease fitness and survival than did the gut microbes in the formula diet group. The results indicated that better prerelease diet training with more appropriate training diets and times could improve the reintroduction of Yangtze sturgeon by adjusting the prerelease gut microbial community. Because a relationship between diet (preference) and gut microbes is common in animals from insects (such as *Drosophila melanogaster*) to mammals (such as *Homo sapiens*), our hypothesis verified by the case study on Yangtze sturgeon applies to other animals. We therefore encourage future studies to identify optimal training diets and times for each species to best adjust its prerelease gut microbial community and then improve its postrelease fitness and survival in reintroduction projects.

## Introduction

With increasing human activities, biodiversity has decreased globally (Butchart et al., 2010; Pimm et al., 2014), which has impacted Earth’s ecosystems and human well-being (Cardinale et al., 2012; Hooper et al., 2012; Oliver et al., 2015). Currently, actions to restore biodiversity are being implemented globally (Mace et al., 2018). Reintroduction, which is the translocation of individuals to areas in which a species has been extirpated with the aim of re-establishing a self-sustaining population, has become a globally important form of conservation management used to restore biodiversity (Cochran-Biederman et al., 2015; Brichieri-Colombi and Moehrenschlager, 2016; Taylor et al., 2017; Haase and Pilotto, 2019; Jourdan et al., 2019). Reintroduction projects have been frequently conducted for many taxa, including mammals, birds, reptiles, fish, and invertebrates (Tavecchia et al., 2009; Dincǎ et al., 2018; Yang et al., 2018; Brichieri-Colombi et al., 2019; Jourdan et al., 2019; Teitelbaum et al., 2019). To date, over 1200 species have been reintroduced (Brichieri-Colombi et al., 2019).

Reintroduction projects require viable source populations for release, derived from either wild or captive-bred populations (Brichieri-Colombi et al., 2019). However, declines in abundance and occurrence have rendered remaining wild populations too fragile to act as continuous sources (Todd and Lintermans, 2015; Brichieri-Colombi et al., 2019), so captive-bred populations are the only choice for reintroduction projects. Captive breeding provides assurance against species extinction and an increased ability to target a specific sex or age cohort for release (International Union for Conservation of Nature [IUCN] and Species Survival Commission [SSC]., 2013; Brichieri-Colombi et al., 2019). However, captive-bred populations often have a relatively low postrelease success in terms of survival, behavior, or breeding performance (Letty et al., 2007; Todd and Lintermans, 2015; Brichieri-Colombi et al., 2019). Short-term postrelease (varying from a few weeks to 1 year in different long-lived species) survival is the crucial first step for the success of reintroduction projects (Svåsand et al., 2000; Armstrong and Seddon, 2008; Tavecchia et al., 2009; Bertolero et al., 2018; Cayuela et al., 2019). Prerelease conditioning has been proven to generally effectively improve short-term postrelease survival (Batson et al., 2015; Tetzlaff et al., 2019). However, starvation is still a significant source of mortality in captive carnivores post release, and foraging deficiencies are underrepresented in the literature (Berger Tal et al., 2019).

Training captive-bred prerelease animals with natural diets could result in increased postrelease survival (Whiteside et al., 2015). Whiteside et al. (2015), indicated that diet conditioning increased postrelease survival by altering foraging efficiency, food discrimination, handling skills, and gut morphology. However, diet preference underlies these four functional effects. Diet preference depends on host gut microbes, which modify host sensory perception (Wong et al., 2017; Yuval, 2017). Therefore, we assume that (i) diet training in captive-bred prerelease animals will determine the drift of host gut microbes, (ii) gut microbes will influence host diet preference, and (iii) diet preference will influence foraging behavior and then impact individuals’ fitness and survival after release.

Yangtze sturgeon (*Acipenser dabryanus*) is a critically endangered endemic species and a flagship species in the Yangtze River (Wu et al., 2014). Because of changes in hydrological conditions driven by human activities, the Yangtze sturgeon has not reproduced naturally since 2000 (Zhang et al., 2011; Wu et al., 2014), and there is currently no natural population in the Yangtze River. Releasing captive-bred populations into appropriate reaches is the only way to restore the natural Yangtze sturgeon population. We retained the F1–F3 generations of a captive-bred population of Yangtze sturgeon and reintroduced more than 100,000 adults and juveniles into the upper Yangtze River from 2007 to 2019. Now, to test our hypothesis and improve our reintroduction programs, we experimentally manipulated early-life diets in the first 7 months after hatching by using natural diets and formula diets for two experimental groups of Yangtze sturgeon and then released some labeled individuals from these two groups into the natural environment. We monitored their body length and weight and gut microbes, and then, by analyzing the two groups’ growth characteristics and gut microbes, we verified the mechanisms by which diet training impacts postrelease fitness and survival.

## Materials and Methods

### Experimental Design and Sampling Procedures

The experimental fish were the F2-generation offspring of a captive-bred population of Yangtze sturgeon. Artificial reproduction of the fish was implemented at facilities of the Yangtze River Fishery Research Institute located in Jingzhou, Hubei Province, China. The offspring came from a single mating pair. A total of 1200 larvae were randomly screened and divided into two groups. Each group was split into three tanks, with 200 fish per tank. The larvae were fed earthworms beginning 8 days post hatching (dph), when the embryonic yolk was completely absorbed. Before feeding, the earthworms were soaked in 2–5 ppm potassium permanganate for 3–5 min, irradiated with UV light for 0.5 h and then minced to an appropriate size for the larvae. When the larvae grew to a full length of 25–35 mm (approximately 23 dph), one group (i.e., natural diet group) continued to feed on earthworms, while the other group (i.e., formula diet group) was gradually transitioned to an artificial formula diet. The duration of the transition period from earthworms to the artificial formula diet was approximately 15 days. Under laboratory rearing conditions, the two treatment groups were fed four times daily (07:00, 13:00, 19:00, and 0:00), fully aerated tap water was used for circulation, and the water temperature and dissolved oxygen content were controlled at 19 ± 0.5°C and 6.5–8.5 mg/L, respectively. Because captive-bred juveniles of Yangtze sturgeon were released in the same year as part of the reintroduction project, 60 individuals of each group were selected randomly, marked, and then released into a natural pond at 7 months post hatching (mph) and recaptured 2 months later (i.e., at 9 mph).

**FIGURE 1:**
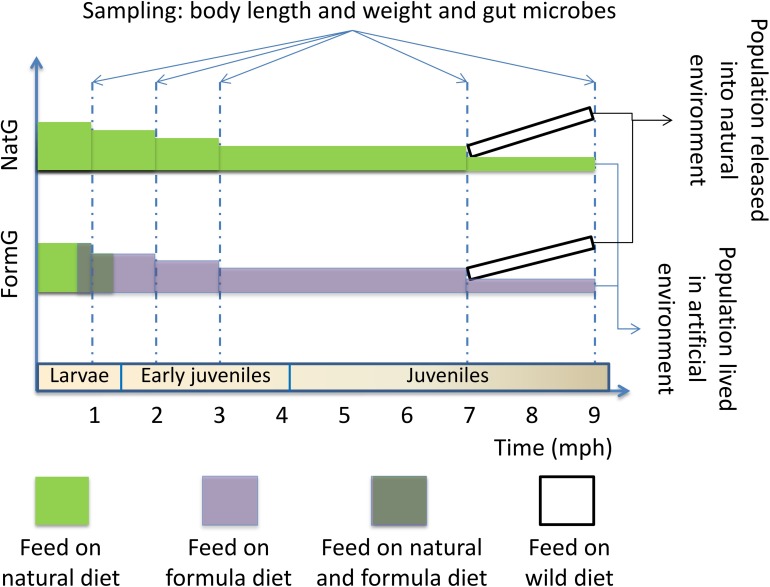
Conceptual diagram showing the experimental procedure. The sampling times and corresponding developmental stages are shown. At 1 mph, the formula diet group was undergoing diet transformation. At 7 mph, some individuals from the two groups were released into the natural environment; the individuals were recaptured 2 months later (9 mph). Sampling includes measurement of body length and weight and extraction of hindgut contents. NatG, natural diet group; FormG, formula diet group; mph, months post hatching.

We randomly sampled individuals from each group five times: larval stage (1 mph), early juvenile stage (2 and 3 mph), and juvenile stage (7 and 9 mph). At the larval stage, the formula diet group was transitioned from earthworms to the formula diet. We measured the body length and weight of the sampled individuals. All sampled individuals were euthanized with an overdose of MS 222 (Sigma, Germany) before dissection, and then, approximately 0.5 g of the hindgut contents was extracted aseptically. The contents from multiple individuals were pooled into one sample, especially for the small individual samples. At least three replicates per group were collected at each sampling time, and the samples were flash-frozen in liquid nitrogen and then stored at −80°C until DNA extraction. The experimental procedure is conceptually outlined in Figure 1.

### DNA Extraction and Sequence Analysis

Microbial DNA was extracted from gut samples using an E.Z.N.A.^®^ Stool DNA Kit (Omega BioTek, Norcross, GA, United States) according to the manufacturer’s protocols. Then, the final DNA concentration and purity were determined by a NanoDrop 2000 UV-vis spectrophotometer (Thermo Fisher Scientific, Wilmington, United States), and DNA quality was checked by 1% agarose gel electrophoresis. The V3–V4 hypervariable regions of the bacterial 16S rRNA gene were amplified with the primers 338F (5′-ACTCCTACGGGAGGCAGCAG-3′) and 806R (5′-GGACTACHVGGGTWTCTAAT-3′) by using a PCR thermocycler system (GeneAmp 9700, ABI, United States). The PCRs were conducted using the following program: 3 min of denaturation at 95°C; 27 cycles of 30 s at 95°C, 30 s for annealing at 55°C, and 45 s for elongation at 72°C; and a final extension at 72°C for 10 min. PCRs were performed in triplicate 20 μl mixtures containing 4 μl of 5 × FastPfu Buffer, 2 μl of 2.5 mM dNTPs, 0.8 μl of each primer (5 μM), 0.4 μl of FastPfu Polymerase, and 10 ng of template DNA. The resulting PCR products were extracted from a 2% agarose gel, further purified using an AxyPrep DNA Gel Extraction Kit (Axygen Biosciences, Union City, CA, United States) and quantified using QuantiFluor^TM^-ST (Promega, United States) according to the manufacturer’s protocol.

Purified amplicons were pooled in equimolar amounts and subjected to paired-end sequencing (2 × 300 bp) on an Illumina MiSeq platform (Illumina, San Diego, CA, United States) according to standard protocols by Majorbio Bio-Pharm Technology Co., Ltd. (Shanghai, China). Raw fastq files were demultiplexed, quality-filtered by Trimmomatic, and merged by FLASH. Operational taxonomic units (OTUs) were clustered with a 97% similarity cutoff using UPARSE (version 7.1)^1^, and chimeric sequences were identified and removed using UCHIME. The taxonomy of each 16S rRNA gene sequence was analyzed by the RDP Classifier algorithm^2^ against the Silva (SSU128) 16S rRNA database using a confidence threshold of 70%. The raw data have been deposited in the CNSA^3^ of CNGBdb with accession number CNP0000907.

### Statistical Analysis

The samples of both groups, including individuals living in the artificial environment and recaptured individuals, from five stages (1, 2, 3, 7, and 9 mph) were delineated into 10 subgroups. Alpha diversity analysis, including analysis of the Chao richness index, Shannon diversity index, and Simpson diversity index, was conducted to reveal the variation in all gut microbial samples. Beta diversity analysis, including hierarchical clustering, sample distances, and non-metric multidimensional scaling (NMDS) analysis, was conducted to reveal the similarity of samples based on their gut microbial communities. The gut microbial community of each subgroup was analyzed at the family level. Analysis of the common OTUs, species, genera, and families between adjacent developmental stages of each group was performed to examine the drift of gut microbial communities with diet training by the natural and formula diets. Analysis of the common OTUs, species, genera, and families between the two groups at each developmental stage was performed to examine the gut microbial community divergence between the natural diet group and the formula diet group. Analysis of the common OTUs, species, genera, and families between each stage and the recaptured stage of each group was performed to examine the fitness of gut microbial communities for release. Then, based on these data sets, (1) lines of best fit for fitness at the OTU, species, genus, and family levels were generated for each group using a polynomial fitting method, and (2) the drift of gut microbial communities was outlined.

## Results

### Growth Characteristics

The growth characteristics of the two groups are shown in Table 1. The body length and weight of the Yangtze sturgeon in the two groups increased continuously from 1 to 9 mph in the artificial environment. The growth characteristics of the individuals in the two groups did not show a significant difference at 1 mph. However, the growth characteristics of the formula diet group at the early juvenile stage (2 and 3 mph) were significantly lower than that of the natural diet group after the diet switch began (*P* < 0.01 and *P* < 0.05). Then, these significant differences vanished as the individuals of the formula diet group adapted to the formula diet at the juvenile stage (7 and 9 mph) (*P* > 0.05). After living in the natural environment for two months (9 mph), the recaptured individuals of both groups exhibited significantly lower growth characteristics than the individuals living in the artificial environment. The recaptured individuals of the natural diet group showed significantly higher growth characteristics than the recaptured individuals of the formula diet group (*P* < 0.05).

**TABLE 1:** Growth characteristics of the natural diet group and formula diet group of *Acipenser dabryanus.*

		**Larval stage**	**Early juvenile stage**		**Juvenile stage**		**Juvenile stage (recaptured)**
		**1 mph**	**2 mph**	**3 mph**	**7 mph**	**9 mph**	**9 mph**
Body length (cm)	NatG	1.83 ± 0.14	6.42 ± 0.65	13.25 ± 0.87	23.76 ± 2.00	29.86 ± 1.65	26.82 ± 2.20
	FormG	2.07 ± 0.13	5.47 ± 0.63	11.72 ± 0.89	23.49 ± 2.72	29.28 ± 1.44	23.89 ± 2.74
	*P*-value	>0.05	<0.01**	<0.01**	>0.05	>0.05	<0.05*
Body weight (g)	NatG	0.08 ± 0.02	2.07 ± 0.56	20.10 ± 3.39	80.22 ± 20.85	152.00 ± 23.62	105.27 ± 27.20
	FormG	0.10 ± 0.03	1.71 ± 0.48	15.14 ± 2.36	92.21 ± 28.11	150.75 ± 20.84	79.56 ± 21.04
	*P*-value	>0.05	<0.05*	<0.01**	>0.05	>0.05	<0.05*

Significance is indicated by * P < 0.05 and ** P < 0.01. NatG, natural diet group; FormG, formula diet group; mph, months post hatching.

Moreover, after 2 months of 120 individuals being released into a natural pond, 37 individuals with natural diet group labels and 32 individuals with formula diet group labels were recaptured. The recapture rate of the natural diet group was 61.67%, and that of the formula diet group was 53.33%. In other words, the survival rate of the natural diet group was 61.67%, and that of the formula diet group was 53.33%.

### Gut Microbes

**FIGURE 2:**
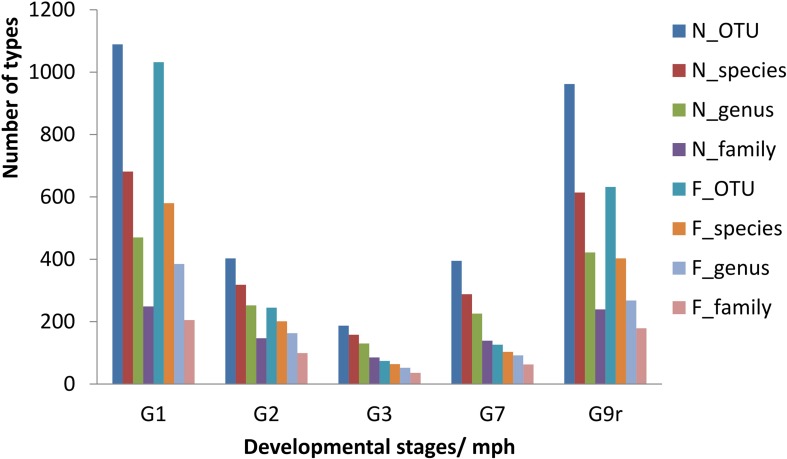
Taxonomic characteristics of gut microbes of the natural diet group and formula diet group of *Acipenser dabryanus* at different stages. G1: the sample subgroup sampled at 1 mph; G2: the sample subgroup sampled at 2 mph; G9r: the sample subgroup sampled from recaptured individuals at 9 mph; N_OTU: natural diet group at the OTU level; F_family: formula diet group at the family level; mph: month post hatching.

A total of 1,406,433 clean sequences were obtained from 38 samples of the two groups, and the average length of these sequences was 437.70 bp. A total of 2165 kinds of bacterial OTUs were detected (UPARSE, 97% cutoff) among these sequences, which belonged to 39 phyla, 84 classes, 192 orders, 351 families, 738 genera, and 1202 species (more details in Supporting Information 1_OTU Table). The OTU counts and compositions of each subgroup were highly variable among sampling times (Figure 2, more details in Supporting Information 2_Subgroup Delineation and Supporting Information 3_Alpha Diversity and Beta Diversity). The dominant taxa at the family level were Clostridiaceae, Enterobacteriaceae, Fusobacteriaceae, Streptococcaceae, Flavobacteriaceae, and Moraxellaceae (more details are provided in Supporting Information 4_Community Heatmap).

### Gut Microbial Community Drift

**FIGURE 3:**
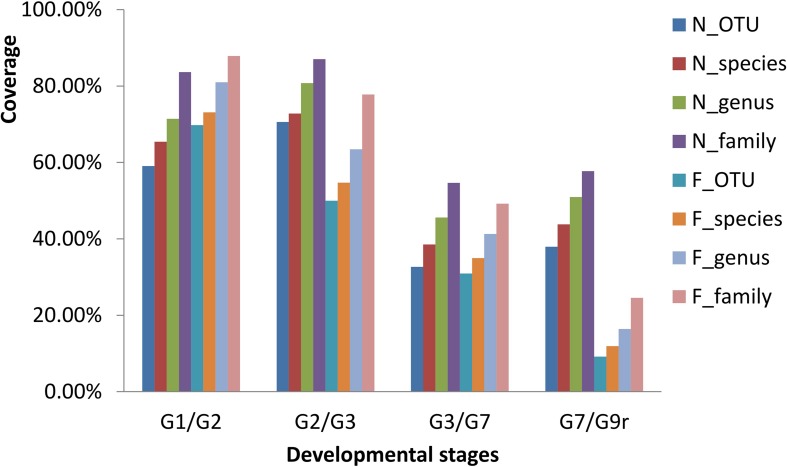
Drift of the gut microbial communities of *Acipenser dabryanus* in the natural diet group and formula diet group. Coverage is the proportion of common OTUs, species, genera and families in two adjacent stages (such as G1 and G2) compared to the later stage (G2). G1/G2: the drift of the gut microbial communities from 1 mph to 2 mph; G2/G3: the drift of the gut microbial communities from 2 mph to 3 mph; G7/G9r: the drift of the gut microbial communities from 7 mph to 9 mph (recaptured); N_OTU: natural diet group at the OTU level; F_family: formula diet group at the family level; mph: month post hatching.

Along with the types of OTUs, the species, genera, and families in the gut microbial communities declined from 1 to 2 to 3 mph (Figure 2), and the contribution rates (indicated by coverage) from the former stage to the latter stage increased in the natural diet group but decreased in the formula diet group (Figure 3). Along with the types of OTUs, the species, genera, and families in the gut microbial communities increased from 3 to 7 to 9 mph (Figure 2), with the contribution rates from the former stage to the latter stage decreasing and then increasing in the natural diet group but continually decreasing in the formula diet group (Figure 3).

**FIGURE 4:**
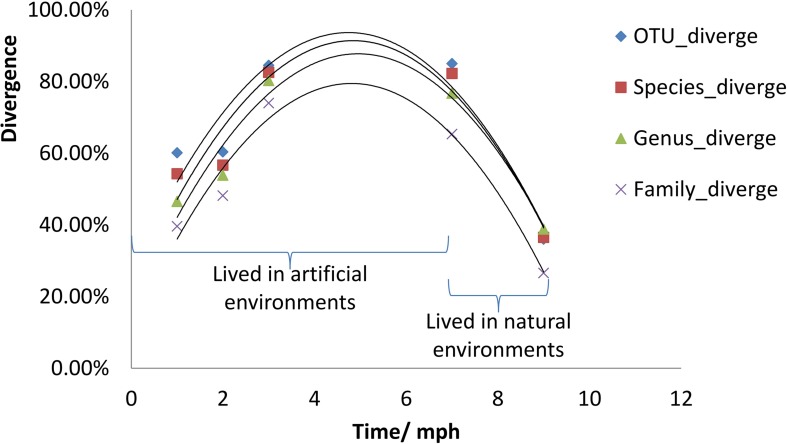
Divergence of gut microbial communities of *Acipenser dabryanus* between the natural diet group and the formula diet group. Divergence is the proportion of total OTUs, species, genera or families unshared by two groups (natural diet group and formula diet group). OTU_diverge: divergence at the OTU level; Species_diverge: divergence at the Species level; mph: month post hatching.

The divergence of gut microbial communities between the natural diet group and the formula diet group increased continually from 1 to 7 mph in the artificial environment (Figure 4). However, the divergence in the natural environment was obviously weaker than that in the artificial environment (Figure 4). In other words, the divergence trend between the two groups was reversed after the individuals were released into the natural environment.

**FIGURE 5:**
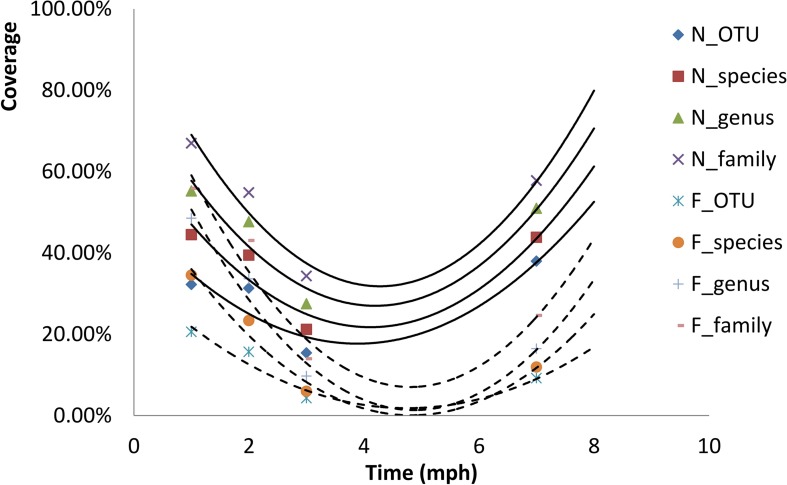
Fitness (coverage) of gut microbial communities of each group of *Acipenser dabryanus at* the OTU, species, genus and family levels at different developmental stages and their lines of best fit. N_OTU: natural diet group at the OTU level; F_family: formula diet group at the family level; mph: month post hatching.

The fitness (indicated by coverage) of the gut microbial communities for release in both the natural and formula diet groups declined continually from 1 to 3 mph, followed by some recovery at 7 mph (Figure 5). The fitness declined more sharply and recovered more weakly in the formula diet group than in the natural diet group (Figure 5). The lines of best fit showed that the fitness of gut microbial communities for release would continue to recover after 7 months of natural diet training following Yangtze sturgeon hatching (Figure 5).

**FIGURE 6:**
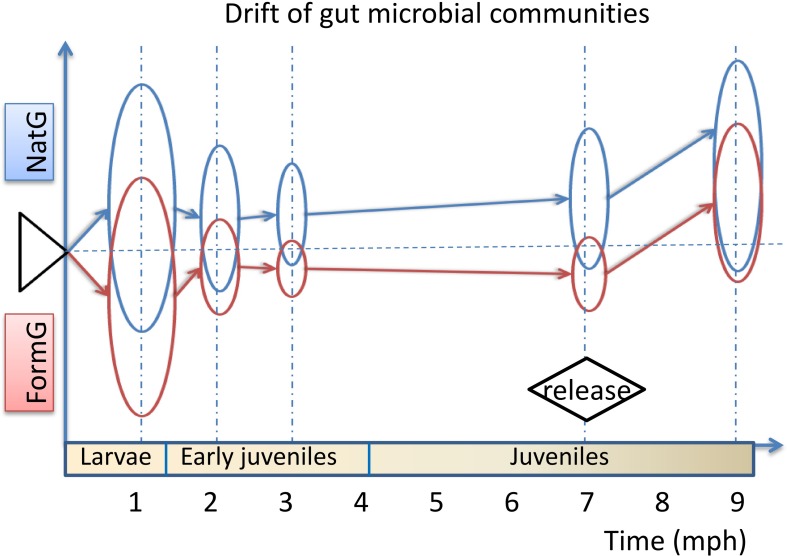
Conceptual diagram showing the drift of gut microbial communities of *Acipenser dabryanus* in the natural diet group and formula diet group at different developmental stages. The ovals represent gut microbial communities. The size of the oval indicates the types of OTU in the gut microbial community of each subgroup. The location of the oval indicates the composition of the gut microbial community of each subgroup. NatG: natural diet group; FormG: formula diet group; mph: months post hatching.

The continually increasing divergence of gut microbial communities between the natural diet group and the formula diet group from 1 to 7 mph in the artificial environment (Figure 4) indicated that the gut microbial communities of the two groups evolved to different statuses. The decreased divergence of gut microbial communities between the natural diet group and the formula diet group at 9 mph in the natural environment (Figure 4) indicated that the gut microbial communities of the two groups began to converge to a common optimal status that fit the natural environment. Because the fitness of the gut microbial community for release in the formula diet group was lower than that in the natural diet group (Figure 5), the optimal status of the gut microbial community in the natural environment was more similar to that of the natural diet group than to that of the formula diet group (Figure 6).

## Discussion

### Diet Training Adjusts Gut Microbes

Diet training changed the gut microbial communities of Yangtze sturgeon individuals. It is well known that diet impacts the types of microorganisms that colonize the gut (Smith et al., 2015; Gajardo et al., 2017; Yang et al., 2019). Generally, the bacterial richness and diversity of developing larvae significantly increase during the first hours, days, or weeks post hatching and then remain stable (Califano et al., 2017; Li et al., 2017; Wilkes-Walburn et al., 2019). As diet is a major contributor to early microbial community development, diet transformation leads to obvious changes in the gut microbial community (Wilkes-Walburn et al., 2019). In the present study, from 1 to 7 mph, the composition of gut microbes varied obviously (Figure 2 and Supplementary Figures SI3_1, 2, 3), and only some microbial types survived to the subsequent stages in both groups (Figure 3), which indicated that the gut microbial communities in each group were drifting. Along with the drift of gut microbial communities, the common microbial types in both groups decreased from 1 to 7 mph (Figure 4), which indicated that the diet training difference drove the gut microbial communities to drift toward different assemblages.

### Gut Microbes Strengthen Diet Preference

Drift of the gut microbial communities influenced Yangtze sturgeons’ preference for a natural diet or formula diet. Gut microbes can impact hosts’ diet preferences and then shape host fitness-related behavior (Alcock et al., 2014; Wong et al., 2017; Akami et al., 2019; Murgier et al., 2019; Patnode et al., 2019). In the current study, at 1 mph, the natural diet group was fed earthworms, and the formula diet group began to transition from earthworms to a formula diet. The mismatch between diet provision and diet preference driven by gut microbes was not very strong, so there were similar growth characteristics between he two groups at 1 mph (Table 1). At 2 and 3 mph, the formula diet group was fed only the formula diet, and the gut microbial communities had not evolved to an optimal assemblage matching the new diet. The mismatch between diet preference and diet provision was substantial, which caused the formula diet group to have significantly lower growth characteristics than the natural diet group (Table 1). After more than 5 months of adjustment, along with the gut microbial communities of the formula diet group evolving to the optimal assemblage for the formula diet, at 7 and 9 mph, the individuals of the formula diet group displayed better growth efficiency, and the previously significant difference in growth characteristics between the natural diet group and formula diet group vanished (Table 1). In other words, under training with different diets, the gut microbial communities in the two groups drifted to different assemblages that supported different diet preferences and matched the different diet provisions. This finding indicated that the gut microbial community impacted diet preference, the mismatch between gut microbial communities and diet provision hampered individuals’ fitness, and the adjustment between gut microbial communities and diet provision supported individuals’ fitness. This finding provides a framework for understanding why 30–40% of the adult individuals of the natural diet group starved to death when fed a formula diet (Du, 2014).

### Prerelease Diet Preference Impacts Postrelease Fitness

Prerelease diet preference impacted the postrelease survival rates and fitness of Yangtze sturgeon. After more than five months of adjustment to diet transformation, at 7 mph, there was no significant difference in growth characteristics between the natural diet group and the formula diet group (Table 1), which indicated that the individuals of the natural diet group were suited to the natural diet and that the individuals of the formula diet group were suited to the formula diet. As the diets provided in the natural environment were more similar to the natural diet than to the formula diet, the individuals in the natural diet group had a higher survival rate and better growth characteristics after release than did those in the formula diet group (Table 1). At the same time, as the diet provisions in the natural environment was different from that in the artificial environment, the released individuals had lower growth characteristics than the artificially fed individuals in both groups (Table 1). This result indicated that the mismatch between prerelease diet preference and postrelease diet provision caused low individual fitness, with greater mismatch causing lower fitness. This is why the survival of reintroduced adults was lower than the survival of adults in the source and control populations and the F1-generation offspring of reintroduced animals survived at rates similar to those of individuals in the source and control populations (Cayuela et al., 2019). The mismatch between the gut microbial community and diet provision was strong enough to have an effect in reintroduced adults but weak enough to go unnoticed in the F1-generation offspring of reintroduced animals.

### The Natural Diet Group Paid a Lower Cost to Adjust Their Gut Microbes to the Natural Environment

To adapt to the natural environment, the formula diet group paid a higher cost for transforming the prerelease gut microbial community into a new optimal assemblage than did the natural diet group. As the gut microbial community impacted host diet preference, to match the diet provision in the natural environment, the gut microbial communities of released individuals needed to gradually evolve into a new optimal assemblage from the prerelease gut microbial community. Because the natural diet was more similar to the diet provision in the natural environment than was the formula diet, the similarity (coverage) of gut microbial communities between prerelease individuals and recaptured surviving individuals was higher in the natural diet group than in the formula diet group (Figure 5). This result indicated that the natural diet group more easily adapted to the environmental change caused by release than did the formula diet group.

### Improvement in Diet Training Depends on the Diet Chosen and Training Time

Better diet training is possible if better diets are chosen with suitable training times. Because the diet provisions in the artificial environment and the natural environment were different, after being released into the natural environment, all individuals needed to adjust their gut microbial communities and diet preferences in order to adapt to the new diet provision. Then, there was an obvious difference in the gut microbial community between prerelease individuals and recaptured individuals (Figure 5 and Supplementary Figures SI3_1, 2, 3). The postrelease mortality rates were 38.33% (in the natural diet group) and 46.67% (in the formula diet group). The growth characteristics of the individuals living in the artificial environment were better than those of the recaptured individuals that had survived post release (Table 1). Using diets that are similar to the diet provision in natural environments to train individuals before release and construct suitable gut microbial communities matching natural environments would be helpful. Because natural populations of juvenile Yangtze sturgeon feed on aquatic oligochaetes, aquatic dipteran larvae, cladocerans, and copepods, and adult Yangtze sturgeon feed on benthic invertebrates and small fishes (Du, 2014), the diet assemblages for training Yangtze sturgeon individuals before release should be more diverse than earthworms. Moreover, a suitable training time that could allow the formation of suitable gut microbial communities is also valuable. For Yangtze sturgeon, because the results showed that the fitness (coverage) of gut microbial communities for release was appropriately 50% after 7 months of natural diet training and would continue to increase after 7 months of natural diet training (Figure 5), we suggest that more than 7 months of natural diet training after hatching would be suitable.

### Improvement in the Reintroduction Effect Requires Improvement in the Source Population With Diet Training

To improve the reintroduction effect, a viable optimal source population is needed. As approximately one-third of postrelease mortality takes place within the first month after release (Tavecchia et al., 2009), short-term postrelease survival is crucial for the success of reintroduction projects (Armstrong and Seddon, 2008; Bertolero et al., 2018; Cayuela et al., 2019). Starvation has been implicated as a significant source of mortality in the first weeks to months in reintroduced captive carnivores (Jule et al., 2008; Kemp and Roshier, 2016; Berger Tal et al., 2019). Better diet training could improve reintroduced individuals’ foraging behavior and thereby improve short-term postrelease survival (Whiteside et al., 2015; Berger Tal et al., 2019). As diet training can adjust hosts’ gut microbes, impact hosts’ diet preference, and then influence short-term postrelease survival rates, for better reintroduction effects, improved source populations with improved diet training are needed. Improved diet training requires diets that are similar to the diet provision in the natural ecosystem and a suitable training time. For Yangtze sturgeon, although prerelease individuals of the natural diet group exhibited high similarity to recaptured individuals at both 1 and 7 mph (Figure 5), we suggest that more than 7 months of natural diet training after hatching is more suitable for release, as populations with larger individuals exhibit a higher survival rate after reintroduction into the natural environment (Sarrazin and Legendre, 2000; Svåsand et al., 2000). Because a relationship between diet (preference) and gut microbes is common in animals from insects (such as *Drosophila melanogaster*) to mammals (such as *Homo sapiens*) (Wong et al., 2017; Akami et al., 2019; Liu et al., 2019; Murgier et al., 2019; Patnode et al., 2019), our hypothesis applies to other animals. Therefore, we encourage further experiments aiming to formulate improved diet training protocols with optimal diets and training times for reintroduction projects of other animals.

## Conclusion

Starvation is a significant source of mortality in captive-bred carnivores after release, and prerelease diet training could improve short-term postrelease survival. Understanding the mechanism of diet training effects is crucial for successful reintroduction projects. In this manuscript, we explore the mechanism by which diet training influences short-term postrelease fitness and survival. Diet training adjusts host gut microbes, which then impact host diet preference. Prerelease diet preference impacts host postrelease fitness, which then impacts postrelease survival rates. Thus, we propose that better diet training leads to a more suitable gut microbial community for release, followed by better postrelease fitness and ultimately better reintroduction effects. Better diet training of a prerelease source population requires (1) the use of better diet assemblages that are similar to the diet provision in natural environments and (2) a more appropriate training time that is long enough to ensure that the host gut microbes form a suitable assemblage for the natural environment.

## Data Availability Statement

The datasets generated for this study can be found in the CNSA (https://db.cngb.org/cnsa/) of CNGBdb with accession number CNP0000907.

## Ethics Statement

The animal study was conscientiously abide by the ethical principles of animal welfare in People’s Republic of China and Hubei Province, accepted the supervision and inspection of the Animal Experimental Ethical Committee of Laboratory Animal Centre, Yangtze River Fisheries Research Institute, Chinese Academy of Fishery Sciences.

## Author Contributions

HY contributed to the conceptualization, formal analysis, visualization, writing the original draft, and reviewing and editing the manuscript. XL worked on the methodology, investigation, data curation, formal analysis, conceptualization, funding acquisition, project administration, supervision, validation, and reviewing and editing the manuscript. HD was responsible for the conceptualization, funding acquisition, project administration, supervision, validation, and reviewing and editing the manuscript. JL and JW carried out the investigation. QW  was responsible for the project administration, resources, supervision, and validation.

## Conflict of Interest

The authors declare that the research was conducted in the absence of any commercial or financial relationships that could be construed as a potential conflict of interest.

## Footnotes

1 http://drive5.com/uparse/

2 http://rdp.cme.msu.edu/

3 https://db.cngb.org/cnsa/

## Supplementary Material

DATA SHEET S1 OTU Table showed the details of gut microbial composition of all samples.

DATA SHEET S2 Subgroup Delineation showed the gut microbial communities and the details of redelineating subgroup.

DATA SHEET S3 Alpha Diversity and Beta Diversity showed the Alpha diversity and Beta diversity of the gut microbial communities of all samples.

DATA SHEET S4 Community Heatmap showed the 50 most abundant families in bacterial communities of each subgroup.
