# Supplementary material for: Adjusting the Prerelease Gut Microbial Community by Diet Training to Improve the Postrelease Fitness of Captive-Bred Acipenser dabryanus
Source: Front Microbiol. 2020 Apr 21;11:488. doi: 10.3389/fmicb.2020.00488 (PMC7186344; doi:10.3389/fmicb.2020.00488)
Supplement: DATA SHEET S2 — Subgroup Delineation showed the gut microbial communities and the details of redelineating subgroup. [file Data_Sheet_2.docx]

Supporting Information 2_Subgroup Delineation

In this case, we randomly sampled individuals from each group five times: larval stage (1 mph), early juvenile stage (2 mph, 3 mph), and juvenile stage (7 mph, 9 mph). Approximately 0.5 g of the hindgut contents was extracted aseptically. The contents from multiple individuals were pooled into one sample, especially for the small individual samples. At least three replicates per group were collected at each sampling time (Table SI2_1).

Table SI2_1 The sample labels of each subgroup in the raw data and in OTU table

| Subgroup_label | Sample_label |
| --- | --- |
| NatG1 | AQY_1, AQY_2, AQY_3 |
| NatG2 | BQY_1, BQY_2, BQY_3 |
| NatG3 | CQY_1, CQY_2, CQY_3 |
| NatG7 | QY_NRW1, QY_NRW2, QY_NRW3, QY_NRW4, QY_NRW5 |
| NatG9r | H_QY_NRW1, H_QY_NRW2, H_QY_NRW3, H_QY_NRW4, H_QY_NRW5 |
| FormG1 | ASL_1, ASL_2, ASL_3 |
| FormG2 | BSL_1, BSL_2, BSL_3 |
| FormG3 | CSL_1, CSL_2, CSL_3 |
| FormG7 | SL_NRW1, SL_NRW2, SL_NRW3, SL_NRW4, SL_NRW5 |
| FormG9r | H_SL_NRW1, H_SL_NRW2, H_SL_NRW3, H_SL_NRW4, H_SL_NRW5 |

A total of 1,406,433 clean sequences were obtained from 38 samples of the two groups at different developmental stages, and the average length of these sequences was 437.70 bp. A total of 2,165 kinds of bacterial OTUs were detected (UPARSE, 97% cutoff) among these sequences. According to analysis of the gut microbial community of the natural diet group at the OTU, species, genus and family levels (Figure SI2_1, Figure SI2_2, Figure SI2_3, Figure SI2_4), we found that sample QY_NRW5 was more similar to samples H_QY_NRW1, H_QY_NRW2, H_QY_NRW3, H_QY_NRW4 and H_QY_NRW5 than to samples QY_NRW1, QY_NRW2, QY_NRW3 and QY_NRW4, which may have been caused by misoperation during sequencing. We placed QY_NRW5 into subgroup NatG9r. According to analysis of the gut microbial community of the formula diet group at the OTU, species, genus and family levels (Figure SI2_5, Figure SI2_6, Figure SI2_7, Figure SI2_8), we found that sample BSL_3 was more similar to samples CSL_1, CSL_2 and CSL_3 than to samples BSL_1 and BSL_2, which may have been caused by misoperation during sequencing. We placed BSL_3 into subgroup FormG3. According to analysis of the gut microbial community of the formula diet group at the OTU, species, genus and family levels (Figure SI2_5, Figure SI2_6, Figure SI2_7, Figure SI2_8), we found that sample SL_NRW1 was more similar to samples CSL_1, CSL_2 and CSL_3 than to samples SL_NRW2, SL_NRW3, SL_NRW4 and SL_NRW5, which may have been caused by misoperation during sequencing. We placed SL_NRW1 into subgroup FormG7. Then, we redelineated the samples of each subgroup (Table SI2_2). **When we did not redelineate the samples, we obtained the same conclusions, although there were small differences in the numerical values of the results.**


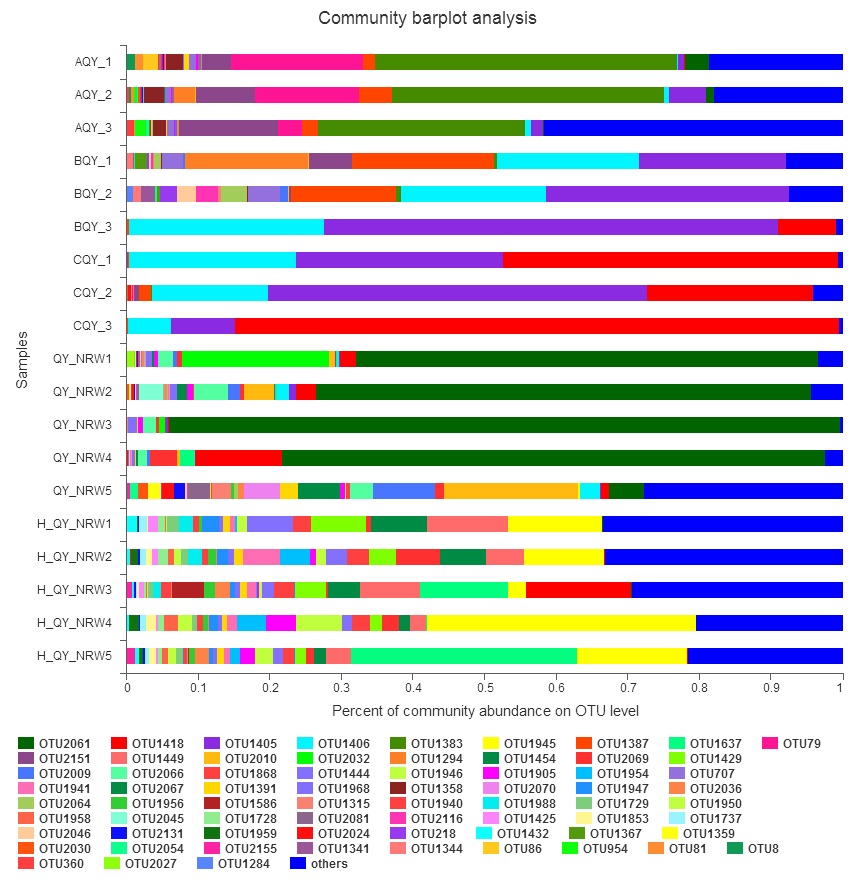


Figure SI2_1 Gut microbial community barplot of the natural diet group at the OTU level


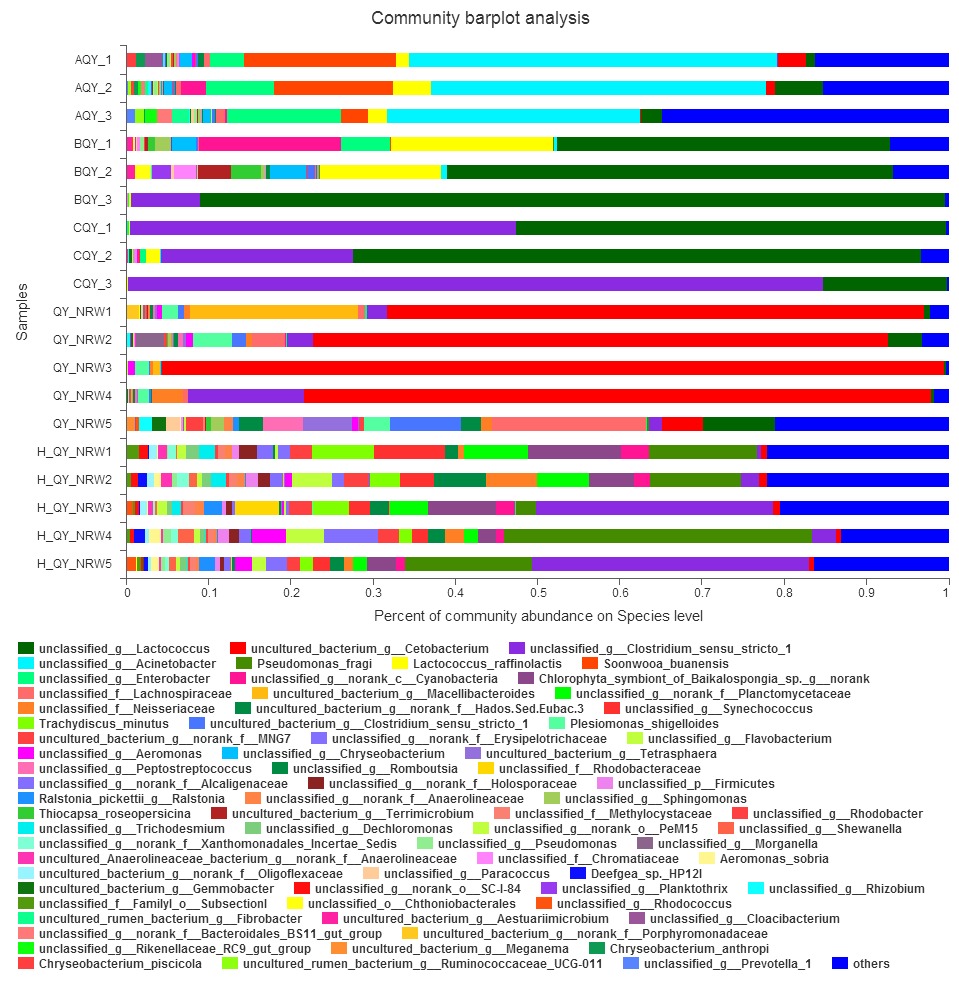


Figure SI2_2 Gut microbial community barplot of the natural diet group at the species level


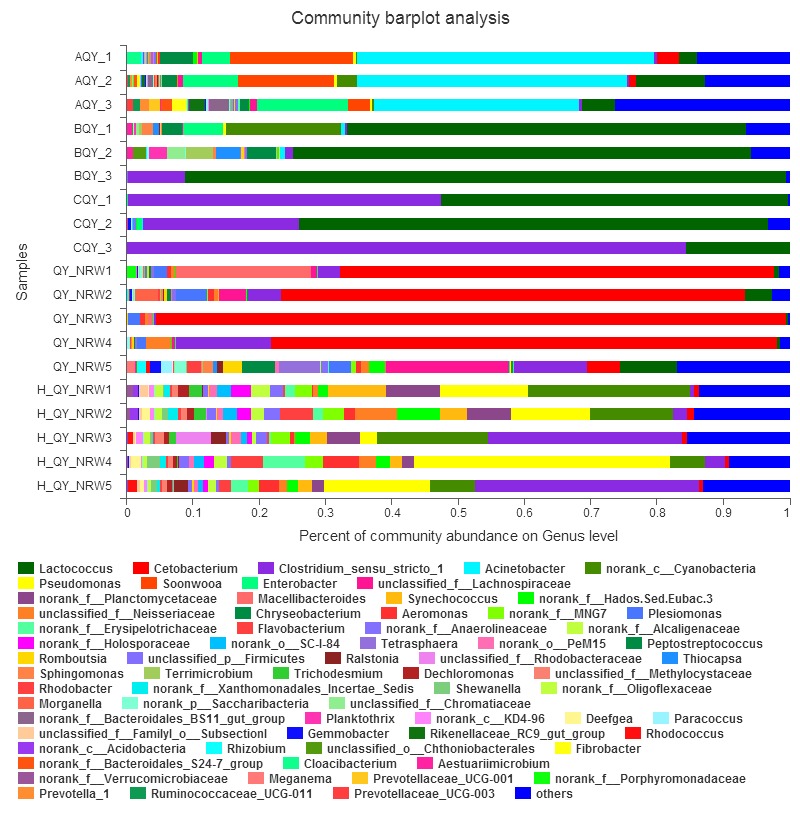


Figure SI2_3 Gut microbial community barplot of the natural diet group at the genus level


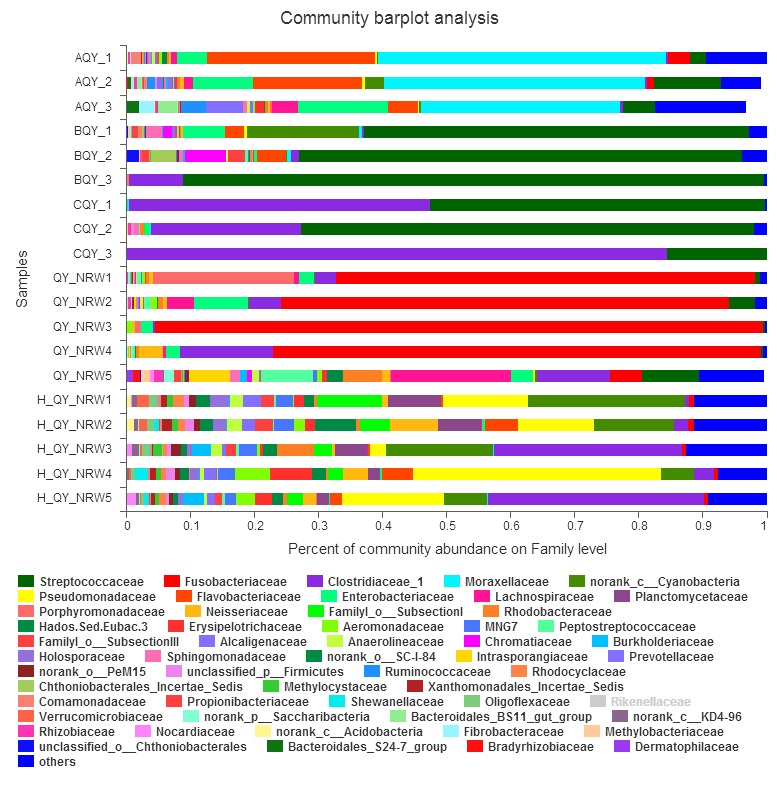


Figure SI2_4 Gut microbial community barplot of the natural diet group at the family level


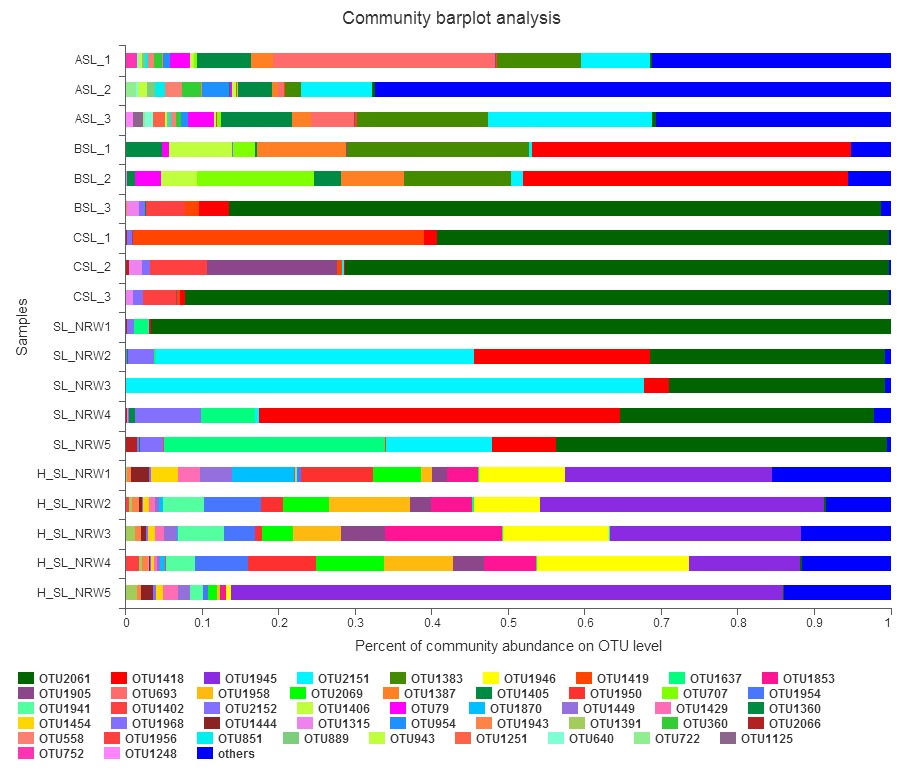


Figure SI2_5 Gut microbial community barplot of the formula diet group at the OTU level


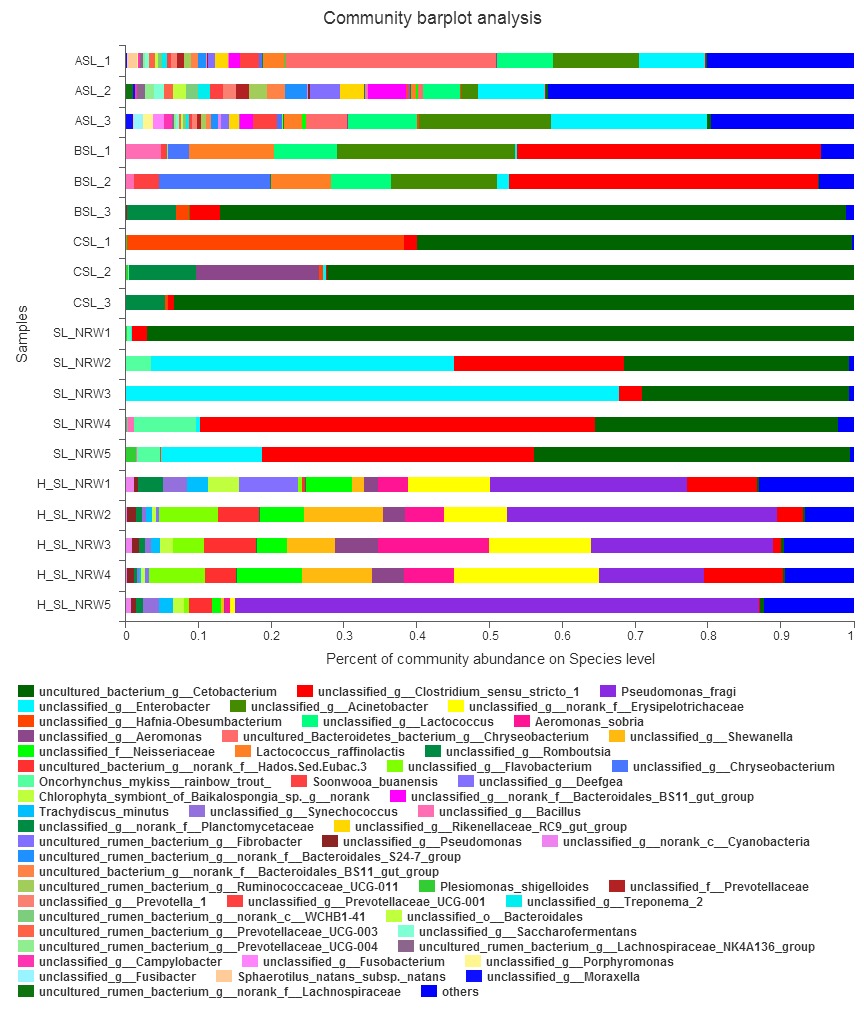


Figure SI2_6 Gut microbial community barplot of the formula diet group at the species level


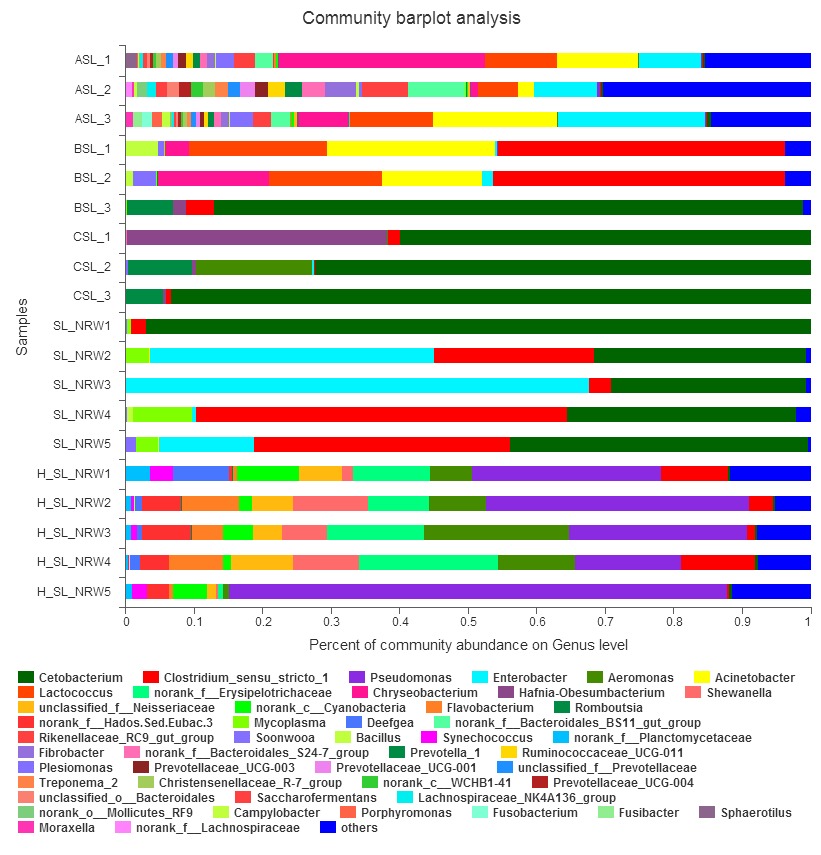


Figure SI2_7 Gut microbial community barplot of the formula diet group at the genus level


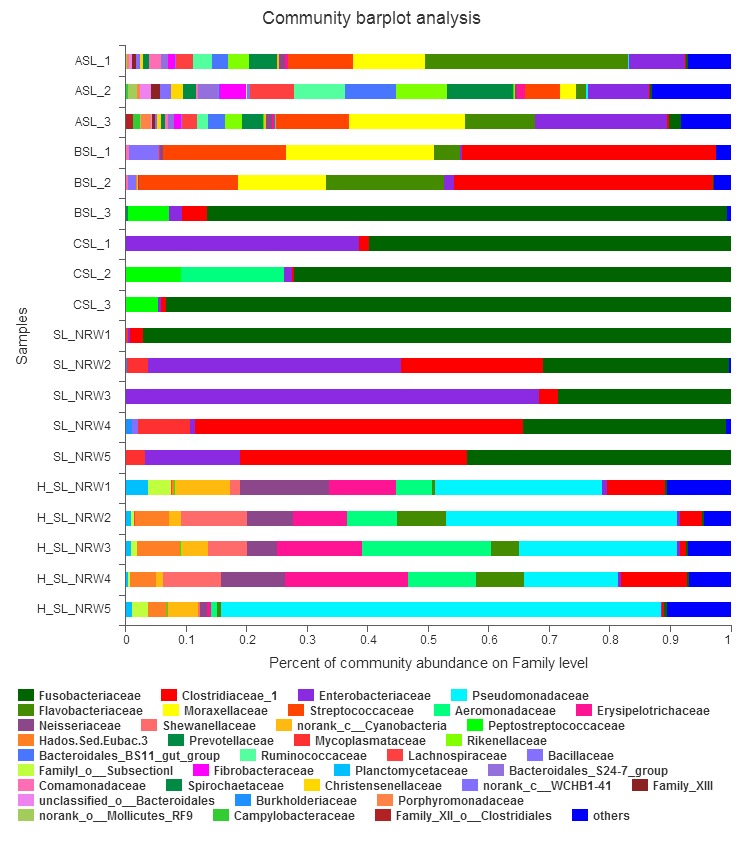


Figure SI2_8 Gut microbial community barplot of the formula diet group at the family level

Table SI2_2 The redelineated samples of each subgroup

| Subgroup_label | Sample_label |
| --- | --- |
| NatG1 | AQY_1, AQY_2, AQY_3 |
| NatG2 | BQY_1, BQY_2, BQY_3 |
| NatG3 | CQY_1, CQY_2, CQY_3 |
| NatG7 | QY_NRW1, QY_NRW2, QY_NRW3, QY_NRW4 |
| NatG9r | H_QY_NRW1, H_QY_NRW2, H_QY_NRW3, H_QY_NRW4, H_QY_NRW5, QY_NRW5 |
| FormG1 | ASL_1, ASL_2, ASL_3 |
| FormG2 | BSL_1, BSL_2 |
| FormG3 | CSL_1, CSL_2, CSL_3, BSL_3, SL_NRW1 |
| FormG7 | SL_NRW2, SL_NRW3, SL_NRW4, SL_NRW5 |
| FormG9r | H_SL_NRW1, H_SL_NRW2, H_SL_NRW3, H_SL_NRW4, H_SL_NRW5 |
