# Supplementary material for: Adjusting the Prerelease Gut Microbial Community by Diet Training to Improve the Postrelease Fitness of Captive-Bred Acipenser dabryanus
Source: Front Microbiol. 2020 Apr 21;11:488. doi: 10.3389/fmicb.2020.00488 (PMC7186344; doi:10.3389/fmicb.2020.00488)
Supplement: DATA SHEET S3 — Alpha Diversity and Beta Diversity showed the Alpha diversity and Beta diversity of the gut microbial communities of all samples. [file Data_Sheet_3.docx]

Supporting Information 3_ Alpha diversity and Beta diversity

In this case, alpha diversity analysis, including analysis of the Chao richness estimate (Figure SI3_1), Shannon diversity index (Figure SI3_2) and Simpson diversity index (Figure SI3_3), was conducted at the OTU level to reveal variation in gut microbes. Beta diversity analysis, including hierarchical cluster analysis (Figure SI3_4), sample distance analysis (Figure SI3_5) and NMDS analysis (Figure SI3_6), was conducted based on Bray-Curtis distances at the OTU level to reveal the similarity and distances of samples in all groups based on their gut microbe compositions.

Here, the subgroup delineation of each sample follows the results shown (Table SI2_2) in Supporting Information 2_Subgroup Delineation.

Table SI2_2 The redelineated samples of each subgroup (from Supporting Information 2_Subgroup Delineation)

| Subgroup_label | Sample_label |
| --- | --- |
| NatG1 | AQY_1, AQY_2, AQY_3 |
| NatG2 | BQY_1, BQY_2, BQY_3 |
| NatG3 | CQY_1, CQY_2, CQY_3 |
| NatG7 | QY_NRW1, QY_NRW2, QY_NRW3, QY_NRW4 |
| NatG9r | H_QY_NRW1, H_QY_NRW2, H_QY_NRW3, H_QY_NRW4, H_QY_NRW5, QY_NRW5 |
| FormG1 | ASL_1, ASL_2, ASL_3 |
| FormG2 | BSL_1, BSL_2 |
| FormG3 | CSL_1, CSL_2, CSL_3, BSL_3, SL_NRW1 |
| FormG7 | SL_NRW2, SL_NRW3, SL_NRW4, SL_NRW5 |
| FormG9r | H_SL_NRW1, H_SL_NRW2, H_SL_NRW3, H_SL_NRW4, H_SL_NRW5 |


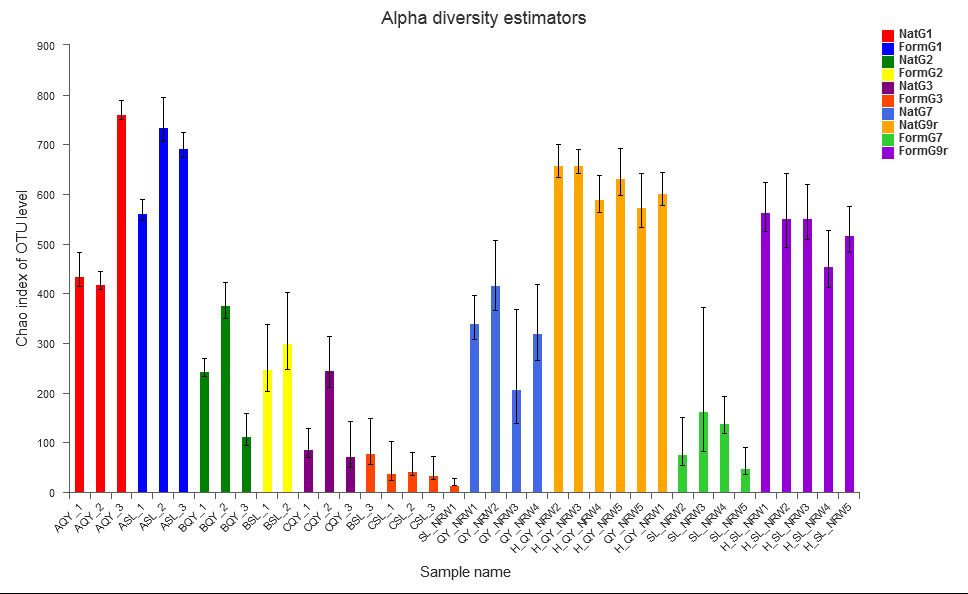


Figure SI3_1 The alpha diversity of the gut microbial communities in each sample indicated by the Chao index at the OTU level. NatG1: the sample subgroup from the natural diet group at 1 month post hatching (mph); FormG1: the sample subgroup from the formula diet group at 1 mph; NatG9r: the sample subgroup from recaptured individuals of the natural diet group at 9 mph.


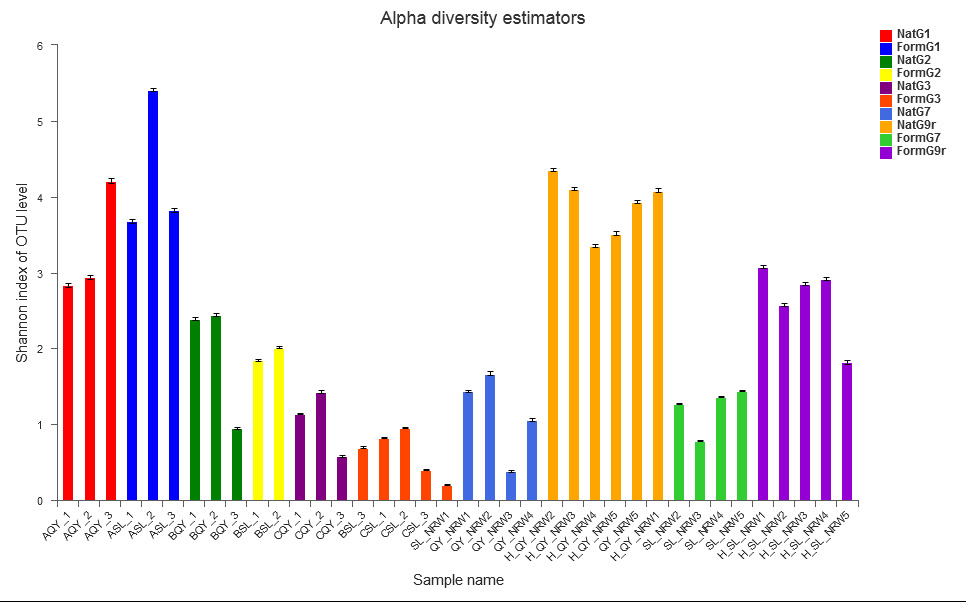


Figure SI3_2 The alpha diversity of the gut microbial communities in each sample indicated by the Shannon index at the OTU level. NatG1: the sample subgroup from the natural diet group at 1 month post hatching (mph); FormG1: the sample subgroup from the formula diet group at 1 mph; NatG9r: the sample subgroup from recaptured individuals of the natural diet group at 9 mph.


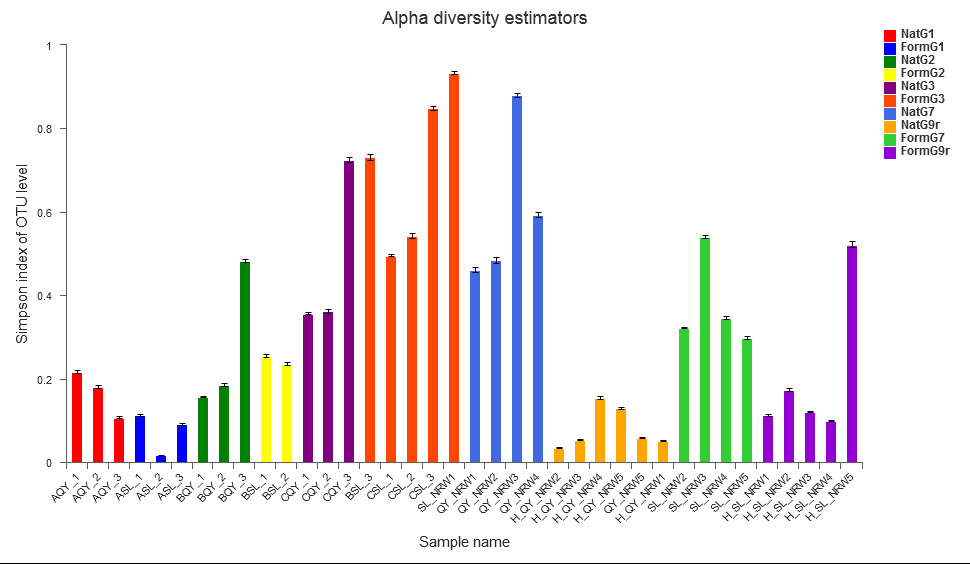


Figure SI3_3 The alpha diversity of the gut microbial communities in each sample indicated by the Simpson index at the OTU level. NatG1: the sample subgroup from the natural diet group at 1 month post hatching (mph); FormG1: the sample subgroup from the formula diet group at 1 mph; NatG9r: the sample subgroup from recaptured individuals of the natural diet group at 9 mph.


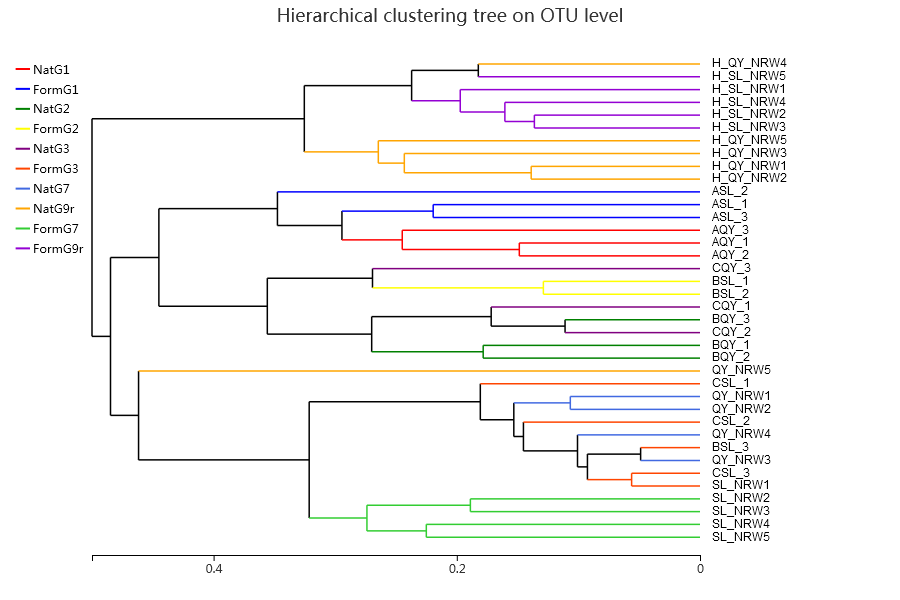


Figure SI3_4 Hierarchical clustering tree of the samples based on the OTU data of their gut microbial communities. NatG1: the sample subgroup from the natural diet group at 1 month post hatching (mph); FormG1: the sample subgroup from the formula diet group at 1 mph; NatG9r: the sample subgroup from recaptured individuals of the natural diet group at 9 mph.


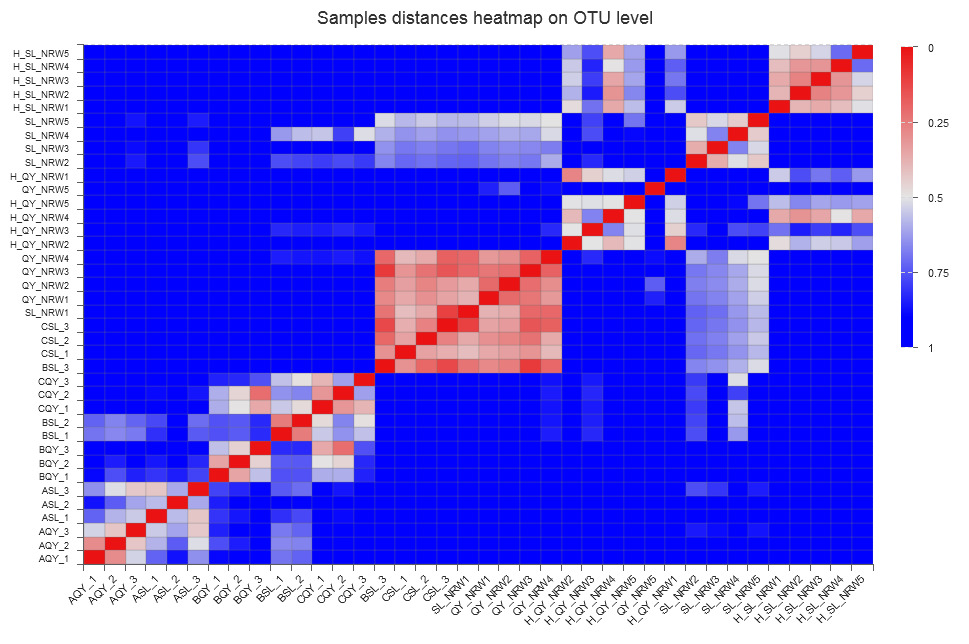


Figure SI3_5 Heatmap showing sample distances based on the OTU data of gut microbial communities.


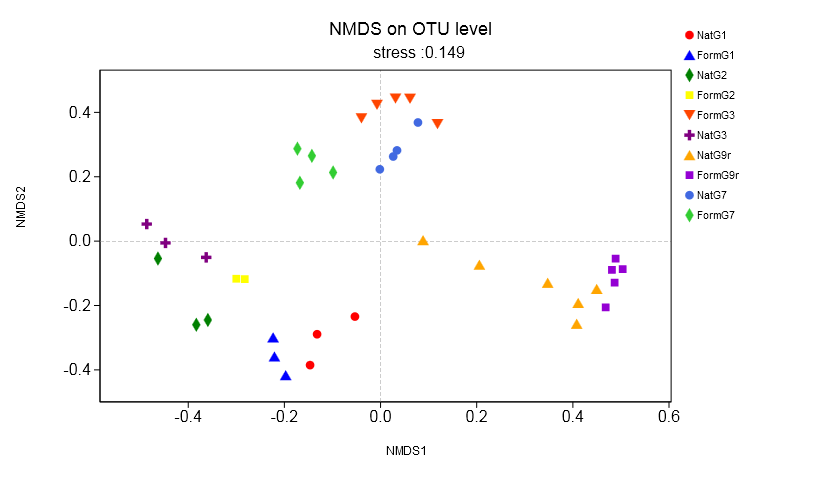


Figure SI3_6 The similarities in the gut microbial communities between samples. NatG1: the sample subgroup from the natural diet group at 1 month post hatching (mph); FormG1: the sample subgroup from the formula diet group at 1 mph; NatG9r: the sample subgroup from recaptured individuals of the natural diet group at 9 mph.
