# Supplementary material for: Adjusting the Prerelease Gut Microbial Community by Diet Training to Improve the Postrelease Fitness of Captive-Bred Acipenser dabryanus
Source: Front Microbiol. 2020 Apr 21;11:488. doi: 10.3389/fmicb.2020.00488 (PMC7186344; doi:10.3389/fmicb.2020.00488)
Supplement: DATA SHEET S4 — Community Heatmap showed the 50 most abundant families in bacterial communities of each subgroup. [file Data_Sheet_4.docx]

Supporting Information 4_Community heatmap

The gut microbial community of each group was analyzed at family level. And then we showed the gut microbial community composition and the abundance of each family in community heatmap (Figure SI4_1).


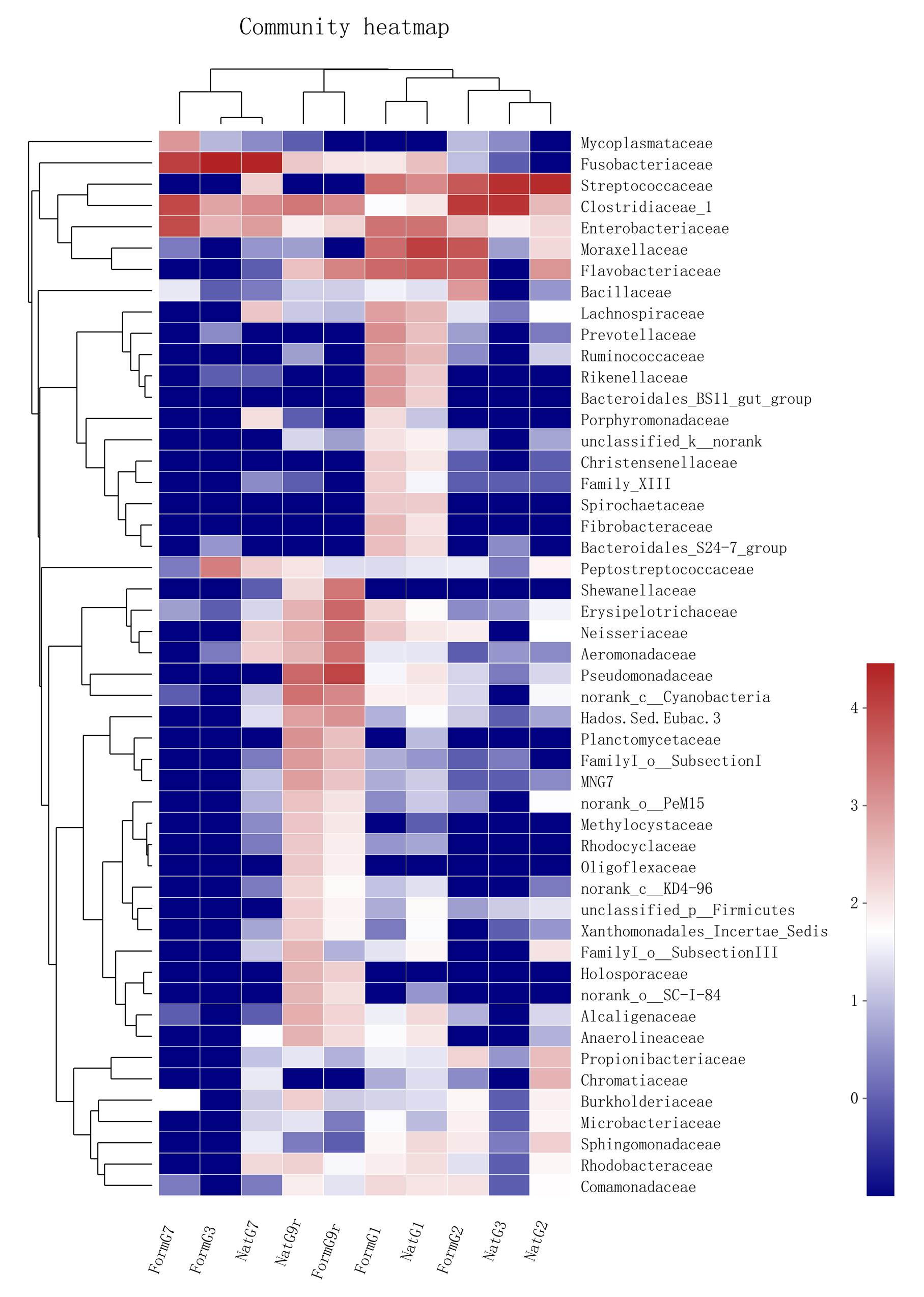


Figure SI4_1 Heatmap of the 50 most abundant families in bacterial communities detected from different developmental stages of *Acipenser dabryanus* feeding on a natural diet (NatG) and formula diet (FormG). Dendrograms of hierarchical cluster analysis grouping families and sample locations are shown on the left and on top, respectively. The color scale represents the normalized values of relative abundances. NatG1: the samples from the natural diet group at 1 mph (months post hatching); FormG1: the samples from the formula diet group at 1 mph; NatG9r: the samples from recaptured individuals of the natural diet group at 9 mph.
